# Bigger is not necessarily better: empirical tests show that dispersal proxies misrepresent actual dispersal ability

**DOI:** 10.1098/rspb.2024.0172

**Published:** 2024-05-22

**Authors:** Jill Lancaster, Barbara J. Downes, Zachary J. Kayll

**Affiliations:** ^1^ School of Geography, Earth and Atmospheric Sciences, The University of Melbourne, , Victoria, 3010, Australia

**Keywords:** aquatic insect, caddisfly, expert opinion, flight, Trichoptera, wing morphology

## Abstract

Tests for the role of species’ relative dispersal abilities in ecological and biogeographical models rely heavily on dispersal proxies, which are seldom substantiated by empirical measures of actual dispersal. This is exemplified by tests of dispersal–range size relationships and by metacommunity research that often features invertebrates, particularly freshwater insects. Using rare and unique empirical data on dispersal abilities of caddisflies, we tested whether actual dispersal abilities were associated with commonly used dispersal proxies (metrics of wing size and shape; expert opinion). Across 59 species in 12 families, wing morphology was not associated with actual dispersal. Within some families, individual wing metrics captured some dispersal differences among species, although useful metrics varied among families and predictive power was typically low. Dispersal abilities assigned by experts were either no better than random or actually poorer than random. Our results cast considerable doubt on research underpinned by dispersal proxies and scrutiny of previous research results may be warranted. Greater progress may lie in employing innovative survey and experimental design to measure actual dispersal in the field.

## 1. Introduction

Dispersal is a fundamentally important ecological phenomenon because it can influence population and community processes, plus the distribution and abundance patterns of species. Dispersal is generally defined as permanent movement away from an origin and settlement at a new location [1] and, therefore, comparatively short-distance, station-keeping movements within the same population are not dispersal *per se* [2]. Dispersal is the main mechanism leading to gene flow among populations [3], is a key component for differentiating different models of metacommunity dynamics [4,5] and is central to the dispersal–range size relationship proposed by biogeographers [6]. Unfortunately, empirical information on actual dispersal abilities required to test such models is difficult to obtain for many organisms and this makes hypothesis tests challenging. These difficulties have rationalized the use of dispersal traits or sets of traits (syndromes). Traits are defined as species-level attributes that ‘...relate to morphological, physiological or life-history features that are inherent to the organism...’ [7, p. 532]. Dispersal traits include attributes such as having wings that allow directed flight or being purely wind-dispersed with less control over direction or distance, for example, ballooning spiders [8]. Recently, the range of dispersal traits has been expanded to include metrics such as body size and whether species are ‘strong’ or ‘weak’ flyers as adjudged by expert opinion, with metric scores compiled into databases (e.g. [9]). We will refer to these metrics as dispersal proxies rather than traits because their correlation with actual dispersal is largely assumed rather than evidence-based. Nevertheless, it follows that the veracity of ecological inferences based on dispersal proxies is contingent upon whether proxies correctly approximate the dispersal process.

Putative dispersal proxies are advocated for use in ecological research (e.g.[ 10]) and particularly in metacommunity research on animal communities, which focuses predominantly on invertebrates and especially those in freshwaters (electronic supplementary material, Appendix S1). Freshwater communities are dominated by aquatic insects, which disperse among systems primarily as flying adults [11]. Accordingly, dispersal proxies of aquatic insects are often related to wing morphology (e.g. wing span [9]), which evidence suggests can reflect some aspects of flight performance [12–14]. Indeed, wing morphology has been used to test hypotheses about insect flight behaviour [15], habitat use [16,17] and distribution patterns [18–20] for both terrestrial and aquatic insects.

A close correspondence between insect wing morphology and dispersal ability is commonly assumed but there are sound reasons why this may be incorrect, including: (i) Dispersal is influenced by numerous, and often inter-related, morphological, physiological, behavioural and ecological factors [3,6,21], and complex ecological processes may not be captured by simple proxies. (ii) Studies of insect flight kinematics and aerodynamics are predominantly laboratory-based, yet flight can differ between artificial and natural environments [22] and, therefore, laboratory studies may not reflect salient field conditions (e.g. weather, vegetation structure, landscape topography). (iii) Wing morphology and other flight attributes may be more closely related to activities other than dispersal, such as load lifting during aerial mating [12,23,24]. Insects fly for many reasons (e.g. true dispersal between populations versus station-keeping movements of foraging, avoiding predators, swarming, oviposition) and multiple selection gradients varying in relative importance could influence wing morphology. Testing the reliability of dispersal proxies empirically is challenging because independent evidence of true dispersal—data that provide unequivocal evidence of dispersal rather than station-keeping movements within a population—is exceedingly rare. One study [25] showed that published data of flying distances ranging from 25 m to >5 km for multiple taxa were a good match to proxy scores within a relevant database. However, flight and dispersal distances are not exactly equivalent, and station-keeping movements cannot be differentiated from dispersal *sensu stricto* in most of the cited studies. To our knowledge, no study has attempted to evaluate dispersal proxies for freshwater invertebrates using data showing actual dispersal (not small-scale movements) in the field. Seemingly broad acceptance of the veracity of dispersal proxies and their widespread use (e.g. [10]) is therefore concerning. A violation of these assumptions could have profound consequences for multiple research fields.

A rare opportunity to test whether, or how, actual dispersal and dispersal proxies are related arose from a recent field survey that quantified instances of dispersal by stream-dwelling caddisflies [26]. Caddisflies have complex life cycles with larvae that are typically confined to aquatic environments and flying adults are the primary means of dispersal among distant or spatially isolated aquatic habitats [11]. Winged adults occur commonly along stream channels in the riparian vegetation [27–29] but may not travel far, and self-recruitment may be significant in many populations (i.e. few individuals disperse and most females oviposit in their natal habitat; examples in Downes *et al*. [30]). True dispersal requires that individuals travel away from natal locations and, for adults, that predominantly means travelling through the terrestrial environment to reach new aquatic habitats. To quantify actual dispersal, Lancaster *et al*. [26] compared the assemblage of adult caddisflies on dry catchment boundaries that were far from permanent water (‘boundary species’) with caddisflies in the riparian zone of permanently flowing water a few kilometres away (‘residents’, which included both boundary and non-boundary species). Individuals on the boundary must have dispersed there because there was no water to support local populations. Two results from that study are germane here.(i) Boundary species were a subset of resident assemblages suggesting that only some species dispersed. (ii) Resident assemblages on opposite sides of catchment boundaries were more similar to one another than to assemblages within the same catchment, suggesting successful trans-catchment dispersal between separate rivers. Thus, boundary species were better dispersers than non-boundary species, but do they differ in dispersal proxies, such as those based on wing morphology or expert opinion?

The primary objective of this study was to test whether the wing morphology of caddisflies is related to empirical, field-based evidence of dispersal, as described above and reported by Lancaster *et al.* [26]. Specifically, we tested whether boundary and non-boundary species differed in aspects of wing morphology known to reflect a capability for long-distance flight. We used the relative abundance of species on catchment boundaries (from [26]) as a measure of relative dispersal ability and tested for associations with wing morphology. For wing morphology, we measured two gross parameters (wing span and area) and two shape parameters (wing aspect ratio (AR) and the second moment of wing area). These metrics reflect aspects of aerodynamic performance according to established models of insect flapping flight [12,13,31] and are described further in §2. Flight performance can be influenced by other, species-specific aspects of wing structure, articulation and kinematics, including among caddisfly species [32–34]. However, measures of size and shape are perhaps the metrics most readily available and easy to calculate, and thus likely to feature in hypothesis tests and to appear in databases of dispersal proxies [9]. There are few tests for associations between wing morphology and ecological attributes of caddisflies, although a positive association between wing size and flight distance has been suggested for some congeneric species [35] and wing morphology may differ among con-familial species depending on whether larvae typically inhabit springs, flowing or standing water [17]. We are unaware of studies encompassing species from multiple families or that provide robust tests for relations between wing morphology and empirical, field-based evidence of actual dispersal. A second objective was to test whether the empirical evidence of caddisfly dispersal (above) corresponded to expert opinion on dispersal ability, as reported in a database for the relevant geographic area [36].

## 2. Methods

### (a) Field sites, survey design and study species

The field survey, described in detail elsewhere [26], involved 10 streams spread across four major catchments in southeastern Australia. This study focussed on nine of those streams. Each stream had three sample locations: Up (on the catchment boundary, i.e. the ridge between two river valleys, but where there was no permanent water), Middle and Down (on the main stem of the river with permanently flowing water).

Flying, adult insects were sampled using light traps (adults of most caddisflies are nocturnal) during the austral summer of 2021 to 2022. The three locations on each stream were sampled contemporaneously over four consecutive nights. Trap design ensured that light projected directly upwards and only attracted insects flying directly overhead so that insects were not attracted from far away. Traps were placed on stream margins at the Middle and Down locations; in Up locations traps were placed at random within the forest in a line roughly parallel to the ridge and just below the ridge line.

A total of 59 species from 12 families collected by Lancaster *et al*. [26] were available and sufficiently numerous for analysis: 39 were boundary species and 20 were non-boundary species (electronic supplementary material, table S2). Analyses featured male specimens only, which maximized the number of species in the analysis because it is impossible to identify females of some species. There was no evidence of strong sex-biased dispersal among these species [26,35] and, although some sexual dimorphism in wing morphology is common in caddisflies [17,35], we assume that morphological patterns among males broadly reflect patterns among females of the same species.

### (b) Wing morphology

**Figure 1.**
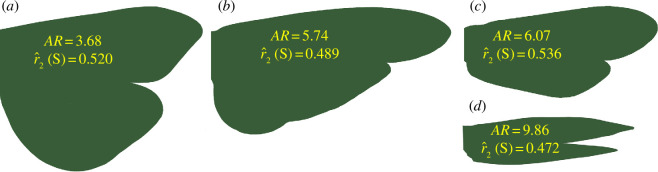
Outlines of coupled fore and hind wings of males of four species of Trichoptera to illustrate variations in shape (not drawn to scale). (*a*) Hydropsychidae: *Asmicridea edwardsi* (McLachlan), (*b*) Leptoceridae: *Triplectides ciuskus ciuskus* (Moseley), (*c*) Ecnomidae: *Ecnomus russellius* (Neboiss), (*d*) Hydroptilidae: *Hellyethira simplex* (Mosely). Shape variables: AR, aspect ratio; ${\hat{r}}_{2}\left(S\right)$ = second moment of wing area.

In flight, the fore and hind wings of caddisflies act as a unified aerodynamic surface during the down stroke [34] and most species have physically coupled wings [37,38]. Thus, caddisflies have four true wings but are functionally two-winged during flight [39]. Accordingly, we calculated wing metrics for coupled wings. Our analysis focused on two first-order descriptions of size—wing area and wing span—and two, non-dimensional, second-order descriptions of shape—wing AR and the radius of the second moment of wing area, ${\hat{r}}_{2}\left(S\right)$ . In general, lift forces increase with wing size but so does mechanical efficiency and the potential for longer flight times. In terms of wing shape (figure 1), high AR reflects slender wings (figure 1*d*), which are associated with power economy and prolonged flight, whereas broad wings have a low AR (figure 1*a*), which may favour slow, agile flight and manoeuvrability [12,15,40]. The second moment of the wing area reflects the force required to generate lift. Values of ${\hat{r}}_{2}\left(S\right)$ are low for wings with broad bases and narrow tips (figure 1*b,d*) and increase as the broadest part of the wing shifts towards the tip (figure 1*a*,*c*). Wings with very broad tips and high ${\hat{r}}_{2}\left(S\right)$ may confer agility and manoeuvrability, but also increase the energetic power required for flight [41]. Conversely, wings with lower values of ${\hat{r}}_{2}\left(S\right)$ (broad bases, or leading and trailing edges that are parallel) may be better suited for extended flight. Thus, hypothetically, good dispersers should have large, narrow wings with broad bases (i.e. high AR and low ${\hat{r}}_{2}\left(S\right)$ ).

One pair of fore and hind wings were removed from each insect, dry mounted on a microscope slide and digitized with a Leica Flexacam C3 attached to a Leica MZ6 stereomicroscope. Wings were arranged in a coupled position, with the forewing span oriented horizontally and perpendicular to the longitudinal axis of the insect body (cf. figure 1). Wing measurements were carried out on images of coupled wing pairs in planform using ImageJ 1.53 t [42] and following calculations described previously [13,35]. There were at least 10 replicates for each species; specimens were sampled at random from resident assemblages. Hair fringes were excluded during measurements for simplicity and because there is no evidence to suggest that hair fringes affect the flight performance of species in our dataset, including the small Hydroptilidae [34]. For miniature insects, which fly at very low Reynolds numbers (*Re*), the area of the hair fringe can exceed that of the wing membrane and influence flight mechanics [43,44]. However, miniature insects are significantly smaller (body length and wing span <1 mm) than even the smallest Hydroptilidae in this study (wing span >2 mm), which fly at much higher *Re*. We did not collect data on body mass to calculate wing loading because the latter metric may be more closely related to load lifting and flight speed than to flight distance [12], and because it is impossible to gather accurate data from field specimens trapped in preservatives (lipids leach rapidly from preserved specimens with a corresponding decline in body mass).

### (c) Proxies based on expert opinion

To meet our second objective, we tested whether dispersal proxies in a database for Australian freshwater invertebrates [36] corresponded to empirical evidence of the dispersal ability of species (reported by Lancaster *et al*. [26]). There are multiple dispersal proxies relevant to insect flight in this database, and scores were largely assigned using expert opinion. One proxy has scores ranging from 0 to 3 (weakest-to-best dispersers), and in the empirical study, we equated a score of 3 with taxa where multiple individuals reached at least one boundary; scores of 2 or 1 with taxa where only one individual reached a boundary; a score of 0 with non-boundary species. Other proxies varied only between 0 (short distance or weak flyers) and 1 (longer distance or strong flyers) and we equated these with non-boundary and boundary taxa, respectively. For each taxon rated in the database, we examined whether the outcomes in the empirical study matched the assigned scores.

### (d) Statistical analyses

Analyses of wing morphology were based on mean values for each of the four wing metrics for each species. Species were replicates in the analyses. For each species, abundance was calculated as the mean number of individuals sampled per trap night, when pooled across Up locations of all streams (data from [26]).

For our primary objective, we first treated dispersal as a categorical variable to test whether dispersal success (i.e. presence on a boundary) was associated with particular wing morphologies. Thus, using a crossed, two-factor design we tested whether wing morphology differed between species grouped by two independent, categorical variables: presence or absence at Up locations (i.e. boundary versus non-boundary species) and taxonomic family (which captured some of the relatedness among species). The four wing metrics were normalized to ensure that each one contributed equally to the analysis and a resemblance matrix was constructed from Euclidean distances between species. This analysis used multivariate permutation tests in PERMANOVA v.7 [45,46].

Second, we treated dispersal as a continuous variable by using variations in the abundance of species at Up locations as a measure of dispersal ability (see Appendix S2 in the electronic supplementary material for rationale). We tested whether dispersal ability could be explained by wing metrics measured for a diverse range of caddisflies, which allowed us to explore variation in dispersal ability in a more sensitive manner than presence/absence on boundaries. We first tested all 59 species together, and then, out of interest, carried out separate tests for each of six families that were sufficiently speciose (≥5 species). This analysis used distance-based linear models (distLM) in PERMANOVA v.7 [45,46], which is analogous to multiple regression. For each of the 59 species, their abundances were log(*X*+1) transformed and converted to a resemblance matrix using Bray-Curtis similarity (electronic supplementary material, Appendix S2). A dummy value of 1 was first added to cells where there were zeroes to enable the calculation of similarities. The wing metrics were inspected with a draftsman’s plot to examine potential correlations and exclude correlated variables if required. Marginal tests examined whether each wing metric individually could explain species’ abundances, and sequential (conditional) tests, which varied the order in which wing metrics were entered into the model, determined which combinations of wing metrics best explained variations in abundance. Akaike information criterion, AIC, was used to determine the best test outcome.

For graphical illustration, we used distance-based redundancy analysis (dbRDA) in which the ordination axes are constrained to be linear combinations of the morphological variables that maximally explain variations among species. Morphological variables were normalized to ensure equal weighting and consistency with the analyses above, and were superimposed onto the dbRDA plot as vectors whose directions and lengths are related to their correlation with the ordination axes and, hence, their role in generating the ordination.

For database proxies, chi-square tests were used to assess whether scores from dispersal proxies differed from random assignment when compared to actual dispersal ability.

## 3. Results

Species’ categorization as boundary or non-boundary species, their relative abundance at Up locations and values for the four wing metrics are presented in electronic supplement material, table S2. Wing size and shape varied greatly among species: the range for mean wing span was 2.3–15.1 mm, area 1–101 mm^2^, AR 3.68–11.5 and ${\hat{r}}_{2}\left(S\right)$ 0.470–0.557 (note that values for ${\hat{r}}_{2}\left(S\right)$ range between 0.4 and 0.7.).

**Figure 2.**
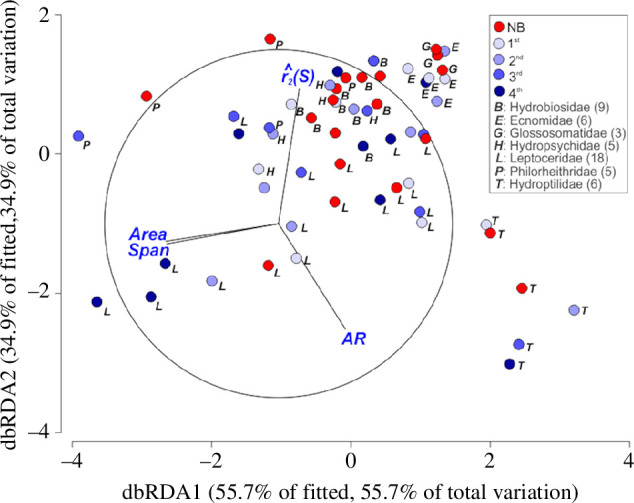
Ordination, dbRDA, relating four wing metrics (*span*, *area*, *AR*, ${\hat{r}}_{2}\left(S\right)$ ) of individual species to caddisfly families (designated by letters), and species' abundances at catchment boundaries (designated by circles of different colours). Only families with at least three species are indicated to reduce clutter. For illustrative purposes, abundances (a continuous variable) have been grouped into bins: NB, non-boundary species; the remaining species were grouped into quartiles based on their abundance, such that 1st, least abundant 25% to 4th, most abundant 25%. Wing metrics are shown as vectors whose direction and length indicate the strength and sign of their correlation with the dbRDA axes. The relative position of the unit circle is arbitrary with respect to the underlying plot.

Caddisfly families differed significantly in wing morphology (PERMANOVA: table 1). The dbRDA ordination clearly shows the separation of most families according to wing morphology (figure 2). The first two ordination axes captured over 90% of the total variation: dbRDA1 was strongly associated with wing size, dbRDA2 with wing shape. Some families, particularly the Hydroptilidae, were separated from all others in ordination space, suggesting a unique wing morphology. Other families, such as the Leptoceridae and Philorheithridae, encompassed a wide and overlapping range of wing sizes, but wing shapes differed systematically between these two families. Specifically, leptocerids had higher AR and lower ${\hat{r}}_{2}\left(S\right)$ than philorheithrids, regardless of wing size. In contrast, the Glossosomatidae and Ecnomidae were clustered close together, suggesting a similar wing morphology for the representatives of these two families. Hydropsychidae and Hydrobiosidae also overlapped greatly in wing morphology.

**Table 1.** Summary of PERMANOVA using four wing metrics to test for differences between taxonomic families and the presence–absence of species on catchment boundaries.

source	df	ms	pseudo-*F*	*p*
presence–absence	1	2.26	1.86	0.199
family	11	11.8	6.25	0.001
p–a × family	7	0.95	0.902	0.902
residual	39	1.89		

The presence–absence of species at catchment boundaries was not associated with wing morphology, nor was there an interaction between family and presence–absence (table 1). Furthermore, inspection of the dbRDA plot suggested no association between family and species' relative abundances on catchment boundaries (see differently coloured symbols with the same letter in figure 2). Most families included non-boundary species (never occurred on catchment boundaries), as well as species with a high abundance. One exception was the three species of Glossosomatidae, which were all non-boundary species, but their wing morphology was broadly similar to species of Ecnomidae and all of the ecnomids were boundary species.

**Figure 3.**
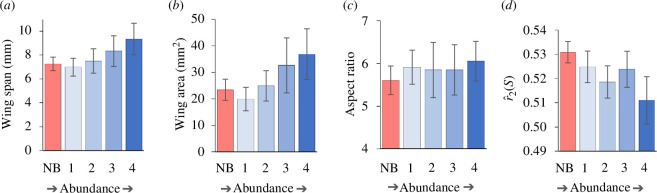
Species in all families categorized according to abundance on catchment boundaries: mean (±s.e.) of two measures of wing size, (*a*) wing span and (*b*) wing area, and two measures of wing shape, (*c*) AR and (*d*) ${\hat{r}}_{2}\left(S\right)$ . See figure 2 for the explanation of abundance groups.

When wing metrics were considered individually, variations in species’ abundances at catchment boundaries (all families and species) were not related to wing size or AR, but there was a weak inverse relationship with ${\hat{r}}_{2}\left(S\right)$ (marginal tests in distLM: table 2, figure 3). Note that wing span was excluded from this analysis because wing span and area were highly correlated and, although both were weakly associated with AR, the area–AR association was weakest. In marginal tests, ${\hat{r}}_{2}\left(S\right)$ was significant and area marginally non-significant but, when variables were combined in sequential tests, only ${\hat{r}}_{2}\left(S\right)$ was significant and wing area was non-significant (table 2). At 9.9%, the variance explained by ${\hat{r}}_{2}\left(S\right)$ was low.

**Table 2.** Summary of the distance-based linear model (distLM) relating species’ abundances on the catchment boundary to wing metrics for all species and for three families with adequate numbers of species and significant results. Wing span was excluded from the analysis (see §3). Outcomes of the best sequential test based on AIC; only significant sequential tests reported. Significant *p*-values in bold; variance explained reported only for significant variables.

taxon	test	variable	pseudo-*F*	*p*	variance explained (%)
all species	marginal	area	2.65	0.087	
		AR	0.822	0.375	
		${\hat{r}}_{2}\left(S\right)$	6.25	**0.012**	9.9
	sequential	${\hat{r}}_{2}\left(S\right)$	6.25	**0.020**	9.9
		area	1.34	0.257	
Leptoceridae	marginal	area	6.55	**0.023**	29
		AR	3.48	0.070	
		${\hat{r}}_{2}\left(S\right)$	0.887	0.349	
	sequential	area	6.55	**0.023**	29
		AR	0.075	0.863	
Hydrobiosidae	marginal	area	1.15	0.330	
		AR	10.8	**0.012**	61
		${\hat{r}}_{2}\left(S\right)$	0.012	0.957	
	sequential	AR	10.8	**0.009**	61
		area	0.324	0.605	
Hydroptilidae	marginal	area	0.144	0.846	
		AR	0.513	0.428	
		${\hat{r}}_{2}\left(S\right)$	3.43	0.057	
	sequential	${\hat{r}}_{2}\left(S\right)$	3.43	**0.046**	46
		area	0.194	0.790	

When the six most speciose families were analysed separately, distLM showed that species’ abundances were related to one wing metric in three families but a different metric in each case (table 2). Abundance at catchment boundaries increased with wing area in the Leptoceridae but increased with AR in the Hydrobiosidae. A negative association between abundance and ${\hat{r}}_{2}\left(S\right)$ was suggested in the Hydroptilidae: this was significant in sequential but not marginal tests, suggesting a weak effect at best. For the remaining three families (Ecnomidae, Hydropsychidae and Philorhethridae), no wing metric was significant in either marginal or sequential tests (electronic supplementary material, table S3). There were, however, rather few species in these families and analyses may have been compromised by small sample sizes (few species) and low inter-species variation, so the results should be interpreted with caution.

All dispersal proxies from the database failed to match empirical evidence of actual dispersal (table 3, electronic supplementary material, Appendix 4). Proxies applied to whole genera or families did particularly poorly (correct scores were significantly less than would be achieved by random assignment) because species within these groups often varied in dispersal ability. Proxies applied to species performed better but outcomes were no better than assigning scores at random.

**Table 3.** Correspondence of scores from five dispersal proxies with empirical evidence of actual dispersal ability for the same taxa, arranged according to taxonomic resolution. Provided are the number of taxa (*N*) with matching scores and a chi-square test of whether the proportion correct is significantly different from random; significant *p*-values in bold (*α* < 0.05). Proxies 3–5 have identical categories but were assigned by different authors and differ in taxonomic resolution. NB = non-boundary species, RB = rarely boundary (only a single individual), B = boundary species. Details of the approach and full results in Appendix 4.

proxy (empirical equivalent)	taxonomic resolution	*n*	*n* (%) with matching scores	% expected if random	*χ* ^2^, *p*‐value
1. dispersal ability (0 = NB, 1 or 2 =RB, 3 = B)	species^a^	14	2 (29%)	25%	no test^b^
	genus + family	10 6	2 (20%) 1 (17%)	25% 25%	no test^b^
	total	30	5 (17%)	25%	
2. females fly <1 km (NB) or >1 km (B) before oviposition	species	21	7 (33%)	50%	2.33, 0.13
	genus + family	12 6	2 (17%) 0 (0%)	50% 50%	10.9, <**0.001**
	total	39	9 (23%)	50%	
3. strong (B) versus weak flyers (NB)	species	21	7 (33%)	50%	2.33, 0.13
	genus + family	12 6	1 (8%) 1 (17%)	50% 50%	11.6, <**0.001**
	total	39	9 (23%)	50%	
4. strong (B) versus weak flyers (NB)	genus	54	19 (35%)	50%	4.74, **0.029**
5. strong (B) versus weak flyers (NB)	family	18	4 (22%)	50%	no test^b^

^a^ Nine species, plus two genera where congeners cannot be separated morphologically, and three genera that each contained one species.

^b^ All taxa assigned the same score.

## 4. Discussion

Documenting actual dispersal in the field is challenging and researchers often rely on proxies of dispersal ability to enable tests of hypotheses in various research areas. For the commonly studied insects, where dispersal is primarily by flying adults, dispersal proxies include some measure of body size or wing morphology. This study used empirical research documenting the dispersal of caddisfly species in the field over ecologically relevant spatial scales reflecting distances that individuals might fly during dispersal between isolated rivers [26]. Contrary to received wisdom, we found little evidence that actual dispersal and wing morphology are correlated. We used two measures of wing size (wing span and area) and two shape parameters (wing AR and ${\hat{r}}_{2}\left(S\right)$ ), and found that they were unreliable proxies for relative dispersal ability within a diverse assemblage of caddisflies. Logically, dispersal and long-distance flight require some minimum flight capability and, according to biomechanical and kinematic studies, insect wing morphology is related to flight performance (e.g. [12] and many others). The failure to find clear-cut or strong relationships in this study, and others [47], suggests other processes affect dispersal outcomes, including behaviour, physiology, life-history trade-offs and context-dependence [3,6,21]. Alternatively, wing morphology may be more closely related to flight functions other than dispersal, such as station-keeping movements of avoiding predators or locating mates. Moreover, our tests of dispersal proxies based on expert opinion showed that these were also unreliable.

### (a) Diverse taxonomic assemblage

Wing morphology was phylogenetically conserved at the family level with strong morphological differences between families, but there were no clear-cut relationships between overall wing morphology and dispersal ability. Instead, all but one of the families had some species that dispersed. Phylogenetic clustering of wing shapes and sizes is a product of evolution and may reflect differences between coarse taxonomic groups (families, orders) in flight strategies [12]. Such patterns have been reported among insect orders [48] and among families within some orders, including the Trichoptera [27]. We failed to find an increase in dispersal ability with wing size (span or area) in the diverse assemblage, nor was there strong evidence for an association with wing shape. We observed only a weak negative effect of ${\hat{r}}_{2}\left(S\right)$ on abundance at catchment boundaries, which is consistent with the suggestion that the energetic power required for flight increases with ${\hat{r}}_{2}\left(S\right)$ [41]. This was, however, a small effect (it explained <10% of the variance in the data), and it would be unwise to use this as a proxy measure of dispersal ability in diverse caddisfly assemblages without further investigation.

### (b) Within families

In contrast to tests encompassing multiple families, there was evidence of associations between individual wing metrics and species' relative dispersal ability within some families. Thus, wing metrics can detect some differences in dispersal ability among some closely related species. Interestingly, the significant metric differed among families. The strongest effect was in the Hydrobiosidae, where AR explained 61% of variation in dispersal ability. Wing area explained 29% of the variation in dispersal ability in the Leptoceridae, and ${\hat{r}}_{2}\left(S\right)$ was weakly associated with dispersal ability in the Hydroptilidae. No significant associations were observed in three other families (Ecnomidae, Hydropsychidae and Philorheithridae), although previous work on some congeneric species of Ecnomidae indicated that wing size, but not shape, was associated with greater dispersal distance [35]. No, or weak, associations between wing morphology and dispersal may be a consequence of small sample size (only five or six species each in these families) and hence low statistical power, but this could also reflect real outcomes. For example, the hydropsychids and hydrobiosids have very similar wing morphology, but tests for species of Hydropsychidae were not even close to significant for any metric. It is feasible that some hydropsychids disperse only rarely. As mentioned earlier (§1), successful dispersal is not simply a reflection of physical capability to disperse but also of behavioural choices. Thus, the suggestion that actual dispersal and wing morphology are associated within some families is tantalizing, but it would be unwise to use these metrics as proxies for dispersal ability without further investigation. Tests involving more species per family are required to determine the veracity of some of these relationships and the utility of any metrics (alone or in combination) as dispersal proxies.

These within-family results suggest that future studies on wing morphology–dispersal relationships should focus on species within a narrow phylogenetic range (i.e. species with the same flight strategy and shared evolutionary history) and thereby minimize the possibility that unmeasured traits might confound interpretations. Indeed, studies that demonstrate inter-specific associations between wing morphology and flight behaviour of insects typically focus on small taxonomic groups, such as one or two families or tribes [15,40] or even variations within species [49].

### (c) Expert opinion

Our results also bring into question the veracity of dispersal proxies based on expert opinion regarding ‘strong’ or ‘weak’ flyers or dispersal affinity scores, especially for groups that differ in taxonomic resolution. All proxies performed poorly and did not reflect actual dispersal abilities that were based on empirical measurements in the field [26]. Matches were very poor for coarse taxonomic groups. Matches improved for species-level identification but were still not better than assigning scores at random. Using expert opinion to assign dispersal scores to coarse taxonomic groups predominates in all of the databases we examined [9,36,50,51], despite at least two obvious problems. First, all these databases use the term ‘traits’ to describe these dispersal measures, but traits are species-level characteristics [8,52] and can also vary within populations [53]. It is unsurprising that species in the same genus or family have different dispersal abilities. As such, while it is possible for traits to be conserved at the genus or even family level, this needs to be demonstrated, rather than assumed. Without such evidence, coarse taxonomic resolution will blur patterns [7] and often deliver misleading findings [54]. Second, the opinions of experts are subject to various cognitive and motivational biases that inevitably result in unreliable information, unless opinions are gathered under strict protocols, that is, soliciting expert opinion, like any method of data acquisition, must be performed rigorously and according to best practice [55]. None of the databases we examined provide information about how expert opinions were gathered or the steps taken to avoid bias. It seems likely that further tests of dispersal scores based on expert opinion in other databases will also find poor correspondence with actual dispersal.

### (d) Broader implications

The implications of these findings go beyond insects and wing morphology. If putative dispersal proxies, including those in databases, are unreliable then hypothesis tests exploiting such information may be unreliable also. The dispersal–range size relationship has received considerable attention (including studies on aquatic insects) but mixed empirical support [6,56], which may relate to the use of proxies that do not reflect actual dispersal. For metacommunity research, hypothesis tests about the role of dispersal commonly focus on freshwater macroinvertebrates and routinely employ proxies to categorize species’ dispersal ability (e.g. [10]). Even coarse descriptors such as active versus passive dispersers (e.g. via advection or zoochory) may provide inconsistent interpretations because both can exhibit similar distribution patterns [19], as can winged and wingless insects [57]. Moreover, freshwater invertebrate communities are extremely diverse taxonomically, potentially encompassing species from multiple phyla, as well as multiple orders and families of insects. Our failure to identify relationships between actual dispersal and dispersal proxies within a single insect order suggests that assuming such relationships exist across even more diverse sets of species is problematic. We note that the common claim that bigger size equates with better dispersal ability among active dispersers (e.g. [9]) often relies on two studies that documented empirical relations between dispersal and body mass [58,59]. However, these two studies used taxa that ranged from bacteria to large mammals (e.g. sperm whales) and body sizes that spanned 10–12 orders of magnitude. Over much smaller ranges in body sizes—such as those found in freshwater macroinvertebrate communities—there was high variability in dispersal ability among taxa and no relation with size, as reported in the original studies [59] and as is apparent in some subsequent investigations, for example, ‘flying insects’ in [60].

New avenues of research may benefit some subject areas that traditionally rely upon assumptions about dispersal ability and dispersal proxies. As a first step, it would be useful to distinguish between traits that reflect within-population, station-keeping movement and traits associated with between-population dispersal, because not all movements constitute dispersal. Distance moved, measured in metres or kilometres, is insufficient to separate these kinds of movements because station-keeping movements may occur over seemingly long distances if a single population covers a large area or long stream length. Conversely, comparatively short-distance movements may constitute true dispersal if otherwise isolated water bodies are geographically close together, for example, adjacent catchments. Species that appear to be ‘poor’ fliers may be good dispersers, for example, if adults are long-lived and can disperse via multiple short flights or zoochory [61]. Insightful lines of future research on dispersal may entail measuring dispersal directly and in natural contexts. Such work is logistically challenging but possible for multi-species assemblages [26,62]. The current study arose from a survey that exploited landscape structures to provide unequivocal evidence of dispersal in the field [26]. Other researchers have also exploited environmental features that allowed direct measurements of dispersal ability in field surveys [63–65], particularly when monitoring the recolonization of new or restored streams [66], and experimental tests of dispersal in the field are possible [67]. Thus, innovative experimental and survey designs may be the keys to success.

## Data Availability

Data are provided in the electronic supplemental material [68].
